# Dose-Sparing Intradermal DTaP-sIPV Immunization With a Hollow Microneedle Leads to Superior Immune Responses

**DOI:** 10.3389/fmicb.2021.757375

**Published:** 2021-10-25

**Authors:** Weilun Zuo, Jingyan Li, Wenwen Jiang, Mengyao Zhang, Yan Ma, Qin Gu, Xiaoyu Wang, Lukui Cai, Li Shi, Mingbo Sun

**Affiliations:** ^1^Yunnan Key Laboratory of Vaccine Research and Development on Severe Infectious Diseases, Institute of Medical Biology, Chinese Academy of Medical Sciences, Peking Union Medical College, Kunming, China; ^2^Laboratory of Vaccine Development, Institute of Medical Biology, Chinese Academy of Medical Sciences, Peking Union Medical College, Kunming, China; ^3^Laboratory of Immunogenetics, Institute of Medical Biology, Chinese Academy of Medical Sciences, Peking Union Medical College, Kunming, China

**Keywords:** hollow microneedle, dose-sparing, DTaP-Sabin IPV, Th1/Th17 response, intradermal immunization

## Abstract

Dose-sparing intradermal (ID) vaccination may induce the same immune responses as intramuscular (IM) vaccination, which can increase vaccine supplies and save costs. In this study, rats were immunized with fractional-dose of Sabin-derived IPV combined with diphtheria-tetanus-acellular pertussis vaccine (DTaP-sIPV) intradermally with hollow microneedle devices called MicronJet600 and the vaccine immunogenicity and efficacy were evaluated and compared with those of full-dose intramuscular immunization. We tested levels of antibodies and the subclass distribution achieved *via* different immunization routes. Furthermore, gene transcription in the lung and spleen, cytokine levels and protection against *Bordetella pertussis (B. pertussis)* infection were also examined. The humoral immune effect of DTaP-sIPV delivered with MicronJet600 revealed that this approach had a significant dose-sparing effect and induced more effective protection against *B. pertussis* infection by causing Th1/Th17 responses. In conclusion, ID immunization of DTaP-sIPV with the MicronJet600 is a better choice than IM immunization, and it has the potential to be a new DTaP-sIPV vaccination strategy.

## Introduction

In the final stage of polio eradication, for the purpose of completely eliminating vaccine-associated paralytic poliomyelitis (VAPP) and disease caused by circulating vaccine-derived polioviruses (cVDPVs), the World Health Organization (WHO) called for global sequential removal of Sabin serotype 2 from the trivalent oral poliomyelitis vaccine (tOPV) and the inclusion of at least one dose of inactivated poliomyelitis vaccine (IPV) in routine infant schedules in 2016 (Manikkam et al., 2013). The Polio Endgame Strategy 2019–2023 requires the urgent introduction of the second dose of IPV to the routine immunizations in 2021–2023 and the use of the full IPV schedule after bivalent oral poliovirus vaccine (bOPV) withdrawal in 2024 (World Health Organization [WHO], 2019). However, only four major manufacturers are currently prequalified by WHO for conventional IPVs (cIPVs), which is produced using virulent poliovirus strains (World Health Organization [WHO], 2015) and another IPV that uses attenuated Sabin strain (sIPV) to avoid the potential biosafety risk of environmental release of virulent polioviruses from cIPV manufacturing facilities was developed recently and manufactured in only China and Japan (Bakker et al., 2011). These factors have led to a global IPV shortage, as the increasing demand cannot be fulfilled by the limited number of manufacturers. Thus, several studies (Schipper et al., 2016; Snider et al., 2019) have investigated intradermal (ID) administration of a fractional inactivated poliovirus vaccine (fIPV), which can induce the same immune effect as full-dose intramuscular (IM) vaccination, to reduce the use of antigens.

In addition, to minimize the number of injections, reduce costs and improve immunization coverage rates, IPV has been used as a combined vaccine together with diphtheria, tetanus, and acellular pertussis (DTP-IPV). Recently, the resurgence of whooping cough despite high vaccination coverage has become a major public health problem worldwide (Cherry, 2012). One major reason for this resurgence is that whole-cell pertussis vaccines (wPVs) were replaced by acellular pertussis vaccines (aPVs) in the 1990s to overcome the serious adverse reactions associated with wPVs. However, the immune responses induced by these two vaccines exhibited some differences. The first generation of pertussis vaccines, wPVs, mainly induced a Th1/Th17 immune response (Higgins et al., 2006; Banus et al., 2008), which plays a major role in the prevention of *Bordetella pertussis (B. pertussis)* infection and the clearance of bacteria (Ross et al., 2013), whereas aPVs skew the immune response to a Th2-type (Mascart et al., 2007), which means suboptimal antibody and T cell responses in eliminating *B. pertussis* (Raeven et al., 2015; Diavatopoulos and Edwards, 2017). In addition, recent research suggests that aPVs induce only waning immunity in children and provide poor protection against transmission in baboons (Klein et al., 2012; Warfel et al., 2014). Therefore, there is an urgent need for new vaccines that can prevent both infection and transmission.

Acellular pertussis vaccines and sIPV are currently administered intramuscularly. Although this immunization route guarantees that the full vaccine dose is delivered, it bypasses the rich network of dendritic cells (DCs), especially Langerhans cells (LCs), presenting in the epidermis and dermis (Glenn and Kenney, 2006). It has been shown that skin antigen-presenting cells (APCs), including DCs, such as LCs in the epidermis and conventional dendritic cells (cDCs) in the dermis, are responsible for activating T cell responses (Klechevsky et al., 2008). In addition, this feature allows some vaccines to induce similar or even stronger immune responses than intramuscular injection after low-dose intradermal injection, and this phenomenon is generally referred to as a dose-sparing effect (Van Damme et al., 2009; Al-Zahrani et al., 2012). Therefore, the intradermal vaccination may be a promising alternative route of administration for *B. pertussis* protection and offers a potential dose-sparing strategy to stretch the limited global IPV supply while further improving population immunity.

Despite many advantages of intradermal immunity, intradermal delivery of vaccines is currently performed using a standard metal needle, which requires significant expertise. The challenge of using a standard needle to directly target the dermis without injecting too deep into the subcutaneous space or leaking vaccine externally, which both frequently occur, has been a major hurdle in expanding the number of vaccines approved for this route of administration (Tarnow and King, 2004). To solve this problem, a hollow microneedle device called MicronJet600 was developed for intradermal injection. It has three pyramid-shaped microneedles with a length of 0.6 mm because this size enables targeting of the vaccine to the dermis with maximal accuracy and minimal experience requirements (Levin et al., 2015).

**FIGURE 1:**
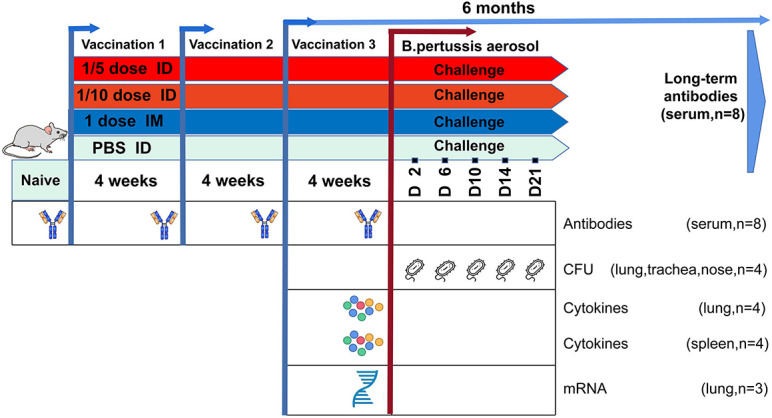
Summary of the study design. Wistar rats were immunized with different doses of DTaP-sIPV by the intradermal (ID; red) or intramuscular (IM; blue) route every 4 weeks. Subsequently, for both routes the vaccination-induced responses were characterized over a period of immunization at four time points including 6 months after the end of immunization for long-term immune-protective observation. In addition, a B. pertussis challenge [10^11^ colony-forming units (CFUs)] was performed on day 0 in all vaccinated groups and control rats (green). Vaccination responses were characterized at the transcriptomic, antibody, and cellular levels on given time points, as depicted.

To the best of our knowledge, there have been no intradermal immunization studies on the Sabin-derived IPV combined with diphtheria-tetanus-acellular pertussis vaccine (DTaP-sIPV) thus far. Therefore, this study was designed to compare the characteristics of the immune response and the vaccine efficacy of full-dose intramuscular injection and low-dose intradermal injection of DTap-sIPV (1/5 and 1/10 of the human dose with the MicronJet600) in rats. We first compared the antibody levels and subclass distribution induced *via* different immune routes and then investigated the gene expression profiles in the lungs and the cytokine profiles and the antibody responses of immunized rats. Finally, we used *B. pertussis* aerosol challenges to evaluate the immune protection induced *via* different immune pathways (Figure 1). Through these findings, we explored whether intradermal injection of a partial dose of DTaP-sIPV can induce an immune response equivalent or superior to that induced by intramuscular injection, whether there is a dose-sparing effect, and whether there is an interaction between antigens when this combined vaccine is delivered *via* the intradermal route.

## Materials and Methods

### Ethics Statement

Equal numbers of male and female Wistar rats (180–220 g) were purchased from Beijing Vital River Laboratory Animal Technology Co., Ltd. All rats were housed in a specific pathogen-free facility at the Institute of Medical Biology, Chinese Academy of Medical Sciences. The institutional Experimental Animal Ethics Committee (approval No. DWSP201911021) approved the experimental protocols.

### Vaccines and Immunization Programs

The Sabin IPV and the DTaP vaccine used in this study was manufactured by the Institute of Medical Biology, Chinese Academy of Medical Sciences (Kunming, China). Three types of Sabin poliovirus (Sabin SO + 1 for type 1, Sabin SO + 1 for type 2, and Pfizer RSO1 for type 3) were used to manufacture for sIPV bulk according with manufacturing protocol as previous described (Liao et al., 2012; Sun et al., 2014). *Bordetella pertussis, Corynebacterium diphtheriae* and *Clostridium tetani* were fermented separately. The components of pertussis antigens Pertussis toxoid (PT), filamentous hemagglutinin (FHA) and Pertactin (PRN) were purified by column chromatography. Purified PT antigen was detoxified by glutaraldehyde and FHA was detoxified by formaldehyde. Diphtheria toxoid (DT) and tetanus toxoid (TT) were obtained from the fermentation harvest of Corynebacterium diphtheriae and Clostridium tetani by clarification, salting out, and detoxification by formaldehyde. Bulk of each component was obtained by adsorption with aluminum hydroxide as adjuvant. The conventional commercial DTaP vaccines contain 25 μg of PT, 25 μg of FHA, 8 μg of PRN, 12.5Lf of DT and 3.5Lf TT per dose and sIPV contain 30DU, 32DU, and 45DU of type 1, 2, and 3, respectively (Nilsson et al., 2005; Jiang R. et al., 2021). The formulations of the vaccines used in this study refer to the components of the conventional commercial vaccines described above. However, since the immunogenicity of DTaP is strong (Boehm et al., 2018), to highlight the effects of immunization routes, the amount of DTaP antigens was reduced proportionally by fourfold in all groups, while for sIPV still uses an undiluted human dose as a full dose. Therefore, the full-dose IM immunity group used a full human dose of sIPV and a quarter human dose of the DTaP vaccine (1D IM group). The corresponding IM immunity group used 1/5 and 1/10 human doses of sIPV and 1/20 and 1/40 human doses of the DTaP vaccine were named as the 1/5D ID group and 1/10D ID group, respectively. The formulation of each dose is shown in Table 1.

**TABLE 1:** Composition of experimental and reference vaccines used in the study.

Antigen concentration	1/5 DTaP-sIPV (1/5D ID)**	1/10 DTaP-sIPV (1/10D ID)**	1DTaP-sIPV (1D IM)*	PBS
Pertussis toxoid (μg/dose)	1.25	0.625	6.25	–
Filamentous hemagglutinin (μg/dose)	1.25	0.625	6.25	–
Pertactin (μg/dose)	0.4	0.2	2	–
Diphtheria toxoid (Lf/dose)	0.625	0.3125	3.125	–
Tetanus toxoid (Lf/dose)	0.175	0.0875	0.875	–
IPV type 1 (DU/dose)	6	3	30	–
IPV type 2 (DU/dose)	6.4	3.2	32	–
IPV type 3 (DU/dose)	9	4.5	45	–
Aluminum hydroxide (mg/dose)	0.15	0.075	0.75	–

*1D IM group contain a full human dose of sIPV and a quarter human dose of the DTaP vaccine.

**The 1/5D and 1/10D represent 1/5 and 1/10 of the antigen doses in the 1D IM group, respectively.

A total of 112 Wistar rats, were randomly assigned into four groups, each group contained 28 rats with an equal number of females and males. Among each group, 20 rats were immunized and challenged with *B. pertussis*, while the others were only immunized and used for long-term protection investigation. The MicronJet600 (Supplementary Figure 1) (Nanopass technologies, Ltd., Israel) was used to inject 100 μl of PBS as the control group or 100 μl of 1/5 dose and 1/10 dose DTaP-sIPV intradermally, while 500 μl of full-dose DTaP-sIPV was injected intramuscularly in the bilateral gastrocnemius muscle. Three doses were given with an interval of 4 weeks (Capeding et al., 2008).

### Sample Collection

Rats were anesthetized (isoflurane/oxygen) for tail vein blood collection and killed by cervical dislocation. The left lung lobes were incubated in RNAlater (Qiagen) and stored at −80°C for RNA sequence. The right lung lobes and trachea, which were used for colonization assays, were homogenized in PBS using a Bio-Gen PRO200 Homogenizer (Pro Scientific), and nose lavages were obtained by flushing the nose with PBS.

Samples of the lungs and spleens were collected in RPMI complete medium (RPMI-1640 medium (Thermo Fisher) supplemented with 10% fetal calf serum (GE Healthcare) and 100 units penicillin and streptomycin) for T cell assays and cytokine detection. Lung tissue was digested with collagenase D (1 mg/ml; Roche) and DNase I (10 mg/ml; Sigma-Aldrich) for 1 h at 37°C with agitation. Next, the lungs or spleens were passed through a 70-mm cell strainer (BD Falcon) to obtain a single-cell suspension, followed by RBC lysis. Whole blood was collected from the tail vein in blood collection tubes 4 weeks after each immunization. For the unchallenged rats in each group, blood was collected 6 months after full immunization. After coagulation (10 min at room temperature), serum was collected by centrifugation (10 min, 3,000 *g*) and stored at −80°C for further use.

### IgG and Subclass Antibody Assay

IgG antibodies and their subclasses, IgG1, IgG2a, and IgG2c, against diphtheria-, tetanus-, and pertussis-related antigens were measured using indirect Enzyme-Linked Immunosorbent Assay (ELISA). Four weeks after each immunization, blood was collected from the tail vein of the rats to separate the serum, and serum antibodies were quantified by ELISA using 3 μg/ml of one antigen: Pertussis toxoid (PT), filamentous hemagglutinin (FHA), Pertactin (PRN), Diphtheria toxoid (DT) or Tetanus toxoid (TT). Bound antibodies were detected using HRP-conjugated anti-rat IgG, IgG1, IgG2a, or IgG2c (SouthernBiotech). Antibody levels were expressed as the geometric mean titer (GMT) (±95% CI), determined by extrapolation of the linear part of the titration curve to two SE above the background value obtained with non-immune rat serum. IgG antibody seroconversion for DTaP antigens was defined as a ≥ 4-fold increase from pre- to post-titer levels.

### Virus Neutralization Assay

Neutralizing antibodies (NAbs) against all three poliovirus types were measured separately according to a previously described method (Sun et al., 2014). Briefly, serum was inactivated at 56°C for 30 min in a water bath. Twofold dilutions of the serum, from 1:8 to 1:16,384, were then incubated for 3 h at 35.5°C with 5% CO_2_ in duplicate with 100 cell culture infective dose 50% (CCID_50_) of the corresponding Sabin-type poliovirus. Hep2 cell suspensions were added, and the mixtures were cultured for 7 days. The results were read macroscopically. An in-house reference serum, whose neutralizing activity was known, was used as a positive control to evaluate the reproducibility of the results. The reciprocal of the highest serum dilution that inhibited 50% of the viral cytopathic effect was taken as the NAb titer against the corresponding poliovirus. Neutralizing antibodies to poliovirus types 1, 2, and 3 were measured with a micro neutralization test according to the method recommended by WHO (World Health Organization, and WHO Expert Committee on Biological Standardization, 2015). The antibody titer ≥ 8 for each polio type was determined as positive conversion, while the fourfold increasing of the antibody levels after immunization was considered as positive conversion if the antibodies were positive before immunization.

### Cytokine Assay

Spleen and lung lymphocytes were isolated as described above (sample collection) 4 weeks after the last immunization. Cells were cultured in complete RPMI-1640 medium supplemented with 10% sterile heat-inactivated fetal bovine serum and 1% penicillin and streptomycin (Thermo Fisher Scientific, United States) at 37°C with 5% CO_2_. For cytokine assay, cells were resuspended at a concentration of 2 × 10^6^/ml and 200 μl were aliquoted into Corning 96-well cell culture flat-bottom plates (Corning, United States) and stimulated with the antigens PT (1 μg/ml), FHA (1 μg/ml), PRN (1 μg/ml). Supernatants were removed after 72 h and stored at −80°C before testing. The cytokine levels were measured using an ELISA kit (Quantikine; R&D Systems) according to the manufacturer’s protocol; interferon gamma (IFN-γ), tumor necrosis factor alpha (TNF-α), interleukin (IL)-2 and IL-6 levels were measured for T helper lymphocyte 1 (Th1) evaluation, IL-4 levels were measured for Th2 evaluation, IL-17A and IL-22 levels were measured for Th17 evaluation, and IL-10 was measured for evaluation of inflammatory restriction as well as the Th2 response (Saraiva and O’Garra, 2010; Raeven et al., 2018, 2020).

### RNA-Sequencing

Total RNA was isolated using RNAzol Bee reagent (Tel-Test Inc.) according to the manufacturer’s protocol. RNA concentrations were quantified using a NanoDrop Spectrophotometer (NanoDrop Technologies) at a wavelength of 260 nm. Oligo(dT)-conjugated magnetic beads were used to purify mRNA. Purified mRNA was fragmented into small pieces with fragment buffer at the appropriate temperature. Then, first-strand cDNA was generated *via* random hexamer-primed reverse transcription, followed by second-strand cDNA synthesis. Afterward, A-Tailing Mix and RNA Index Adapters were added and incubated for end repair. The cDNA fragments obtained from the previous step were amplified by PCR, and the products were purified by Ampure XP Beads and then dissolved in EB solution. The product was validated on an Agilent Technologies 2100 bioanalyzer for quality control. The double-stranded PCR products from the previous step were heated, denatured and circularized by the splint oligo sequence to obtain the final library. Single-strand circular DNA (ssCir DNA) was the format of the final library. The final library was amplified with phi29 to generate DNA nanoballs (DNBs), which contained more than 300 copies of one molecule. DNBs were loaded into the patterned nanoarray, and paired-end 100-base reads were generated on the BGISeq500 platform (BGI-Shenzhen, China).

### Transcriptomic Data Analysis

The sequencing data were filtered with SOAPnuke (v1.5.2) (Li et al., 2008). The clean reads were mapped to the reference genome using HISAT2 (v2.0.4) (Kim et al., 2015). Ericscript (v0.5.5) (Shen et al., 2014) and rMATS (V3.2.5) (Langmead and Salzberg, 2012) were used to fuse genes and differentially spliced genes (DSGs), respectively. Then, the expression level of the gene was calculated by RSEM (v1.2.12). The normalization method used in transcriptomic analysis was FPKM (Fragments Per Kilobase per Million mapped reads). The heatmap was drawn with Heml (v1.0.3.7) according to the gene expression in different samples. Essentially, differential expression analysis was performed using DESeq2 (v1.4.5) with a fold ratio (FR) > 1.5 and *Q* value < 0.05. To gain insight into changes in phenotype, GO^1^ and KEGG^2^ enrichment analyses of annotated differentially expressed genes were performed with Phyper^3^ based on hypergeometric tests. The significance levels of terms and pathways were corrected according to the *Q* value with a rigorous threshold (*Q* value < 0.05) by the Bonferroni test.

### *Bordetella pertussis* Respiratory Challenge

The bacterial strain used for the challenge was pertussis strain BP-L1, which was isolated from clinical cases in Yunnan, China, in 2019. The alleles were ptxA1, prn2, fhaB1, fim3A and ptxP3, consistent with the current epidemic strains. The phylogenetic tree constructed using ptxA, fhaB, prn, and ptxP sequences indicated that BP-L1 formed a clade together with D420, which is a recent clinical isolated from human infants with severe respiratory distress (Warfel et al., 2012; Supplementary Figure 2). *B. pertussis* was grown from a frozen stock on Bordet-Gengou agar plates containing glycerol and 15% sheep blood at 37°C with a diameter of 9 cm. After 3 days of culture, the bacteria were subcultured again on the same Bordet-Gengou plate for 48 h. Bacteria were centrifuged and resuspended in PBS, and the OD was measured at 530 nm. *B. pertussis* infection of Twenty rats, females and males, from each group were performed 4 weeks after the last immunization by aerosol challenge (10^11^ CFU/ml) administered using a nebulizer over 30 min (Jiang W. et al., 2021). As described before, the course of infection was monitored by colony-forming unit (CFU) counts on lung and tracheal homogenates and nose lavage at intervals post-infection (p.i.) (McGuirk et al., 1998).

### Statistical Analysis

Statistical analysis was carried out using GraphPad Prism 7.0. One- or two-way analysis of variance (ANOVA) was used to analyze the statistical significance of differences among three or more groups. *P* values less than 0.05 were considered to be statistically significant. For transcriptome analysis, *P* values were Bonferroni test corrected to obtain *Q* values and *Q* values less than 0.05 were considered to be statistically significant.

## Results

### A Dose-Sparing Effect of at Least Fivefold Was Induced by Intradermal Immunization

**FIGURE 2:**
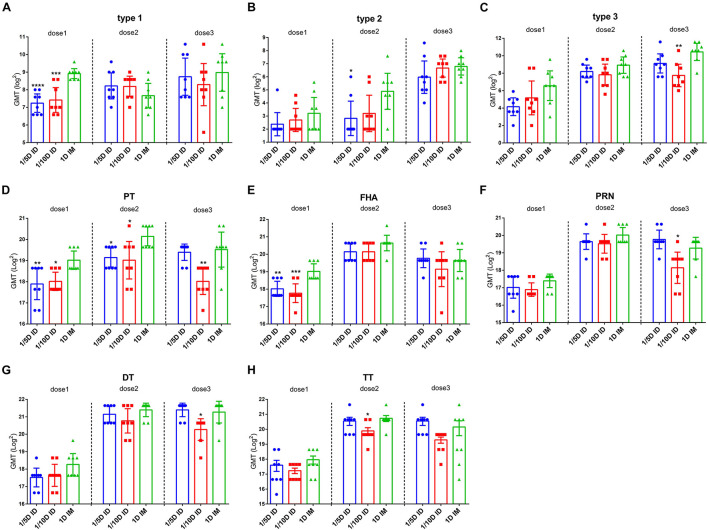
Intradermal immunization induced the same antibody levels as the full-dose of the intramuscular immunization did. ELISAs were used to compare serological responses from rats immunized through 1/5 or 1/10 dose ID to 1 dose IM rats (*n* = 8). Virus neutralizing antibody titers from rats at 28 days after every dose of immunization **(A)** type 1 **(B)** type 2 and **(C)** type 3, respectively. The results were shown as the log2 of virus neutralizing titers of the geometric mean ± 95% CI. Total IgG serum antibody were quantified against **(D)** PT, **(E)** FHA, **(F)** PRN, **(G)** DT and **(H)** TT at 28 days after each dose of immunization. The results were shown as the log2 of antibody titers of the geometric mean ± 95% CI. The PBS group results are below the detection limit and were not shown in figure. Data were analyzed using an one-way ANOVA model with Tukey’s multiple comparisons test. *P < 0.05, **P < 0.01, ***P < 0.001, and ****P < 0.0001 vs 1D IM group (*n* = 8).

To evaluate the immune responses induced by different doses and routes of immunization, antibodies against Pertussis toxoid (PT), Filamentous hemagglutinin (FHA), Pertactin (PRN), Diphtheria toxoid (DT) or Tetanus toxoid (TT) along with the neutralizing antibodies (NAbs) of all three serotypes of polioviruses were measured before vaccination and 28 days after each dose of vaccination (Figure 2).

Before vaccination, all three experimental groups tested negative for all types of antibodies, as did the PBS control group post-vaccination. The results showed a consistent trend of seroconversion rate in all three experimental groups (1/5D ID, 1/10D ID, and 1D IM). The seroconversion rates for PT, FHA, PRN, DT and TT reached 100% after the first dose, those for type 1 and type 3 reached 100% after the second dose, and those for type 2 reached 100% after the third dose (Table 2).

**TABLE 2:** Seroconversion rates against types 1, 2, and 3 poliovirus strains, and PT, FHA, PRN, DT, and TT after vaccinations.

Seroconversion rate (%)		1/5D ID (*n* = 8)	1/10D ID (*n* = 8)	1D IM (*n* = 8)	PBS (*n* = 8)
Type1	Dose1	87.5	100	100	0
	Dose2	100	100	100	0
	Dose3	100	100	100	0
Type2	Dose1	12.5	37.5	50	0
	Dose2	25	50	87.5	0
	Dose3	100	100	100	0
Type3	Dose1	87.5	87.5	100	0
	Dose2	100	100	100	0
	Dose3	100	100	100	0
PT	Dose1	100	100	100	0
FHA	Dose1	100	100	100	0
PRN	Dose1	100	100	100	0
DT	Dose1	100	100	100	0
TT	Dose1	100	100	100	0

Antibody geometric mean titer (GMT) induced by different immunization routes were investigated. After the first vaccination, the GMT for PT, FHA and type 1 of polio virus in the 1/5D ID and 1/10D ID groups were lower than those in the 1D IM group, with no significant differences in the remaining antibodies. After the second vaccination, the titers of antibodies to PT, type 2 polio virus in the 1/5D ID group and antibody to PT in the 1/10D ID group were lower than those in the 1D IM group, with no significant differences in the remaining antibodies. After the third vaccination, the GMT for PT and type 3 polio virus antibodies in the 1/10D ID group were lower than those in the 1D IM group, while the levels of all types of antibodies in the 1/5D ID group were not significantly different from those in the 1D IM group (Figure 2).

**FIGURE 3:**
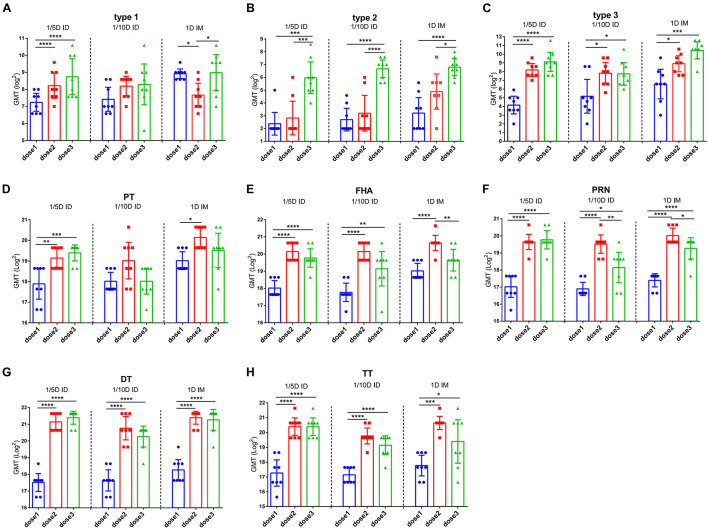
The efficacy of booster immunization in the same group. Poliovirus **(A)** type 1, **(B)** type 2 and **(C)** type 3 neutralizing antibody levels and **(D)** PT, **(E)** FHA, **(F)** PRN **(G)** DT and **(H)** TT antibody levels were compared between the different vaccination in the same group. The results were shown as the log2 of antibody titer of the geometric mean ± 95% CI. The PBS group results are below the detection limit and were not shown in figure. Data were analyzed using an one-way ANOVA model with Tukey’s multiple comparisons test. *P < 0.05, **P < 0.01, ***P < 0.001, and ****P < 0.0001 (*n* = 8).

To determine the efficacy of booster immunization, antibody levels were compared among the different doses in the same group. Both DTaP-related antibodies (PT, FHA, PRN, DT, and TT) and the three types of polio virus NAbs were significantly elevated in all experimental groups after the second vaccination (Figure 3). However, compared to the significant increasing of NAbs after the second dose, the third dose did not increase the levels of any antibody significantly except for the antibody against type 2 poliovirus in the 1/5D ID group. In addition, a significant decreasing in antibodies for FHA and PRN were observed in different groups. This situation was widely observed in different groups (Figure 3). This result indicates that the two-dose immunization program may apply to DTaP-sIPV intradermal immunization while adjusting the dose of type 2 IPV so that the other antigen can be further saved without affecting the antibody levels. In addition, the distributions of IgG2c (Th1-associated) and IgG1/2a (Th2-associated) antibody subclasses are presented as the proportions of total levels (Supplementary Figure 3). The subtypes of antibodies against PT and PRN show a slight difference in the Th1 response in intradermally immunized rats (Mawas et al., 2005).

**FIGURE 4:**
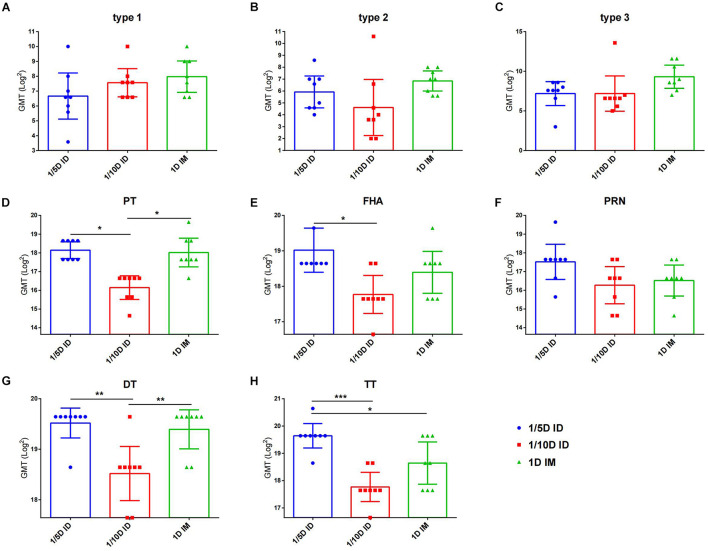
After 6 months of vaccination, intradermal immunization induced the same antibody levels as the full-dose of the intramuscular immunization did. Six months after the last dose of immunization, serum antibody levels in unchallenged rats were examined to reflect the long-term protection induced by different vaccines. Virus neutralizing antibody titers for **(A)** type 1, **(B)** type 2 and **(C)** type 3, respectively. The results were shown as the log2 of virus neutralizing titers of the geometric mean ± 95% CI. Total IgG serum antibody were quantified against **(D)** PT, **(E)** FHA, **(F)** PRN, **(G)** DT and **(H)** TT at 28 days after each dose of immunization. The results were shown as the log2 of antibody titers of the geometric mean ± 95% CI. The PBS group results were below the detection limit and are not shown in figure. Data were analyzed using an one-way ANOVA model with Tukey’s multiple comparisons test. *P < 0.05, **P < 0.01, and ***P < 0.001 (*n* = 8).

To investigate the long-term protection, we continually detected the antibody levels against each vaccine component of unchallenged rats after three doses immunization. At 6 months post-vaccination, no difference for three types of anti-polio among three groups was found. 1/5D ID versus the 1D IM group showed similar antibody levels against PT, FHA, PRN and DT, but higher TT antibody level in the 1/5D ID group than that in the 1D IM group were tested (Figure 4). On the contrary, the antibody against PT, FHA, PRN and DT were lower in 1/10D ID group than either or both of the 1/5D ID and the 1D IM group. In terms of antibody level, 1/5D ID vaccination elicited comparable long-term protection against full-dose intramuscular vaccination.

In general, intradermal DTaP-sIPV immunization showed a significant dose-sparing effect, and three 1/5 doses of DTaP-sIPV administered intradermally with the MicronJet600 induced the same level of humoral immunization as full-dose IM immunization and can last at least 6 months.

### Intradermal Immunization Induced Superior Protection Against *B. pertussis* Infection

To assess protection against pertussis infection, immunized rats were challenged with aerosolized *B. pertussis*, and the pertussis colony-forming unit (CFU) in lung homogenate, trachea homogenate and nasal lavage fluid were counted.

**FIGURE 5:**
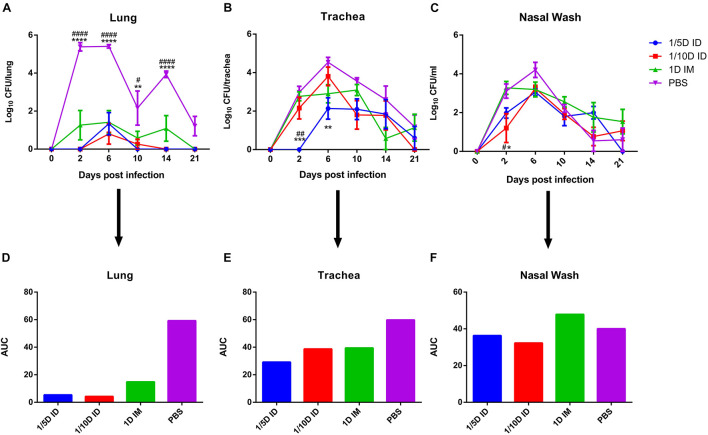
Intradermal immunization induced more effective protection against B. pertussis infection. Protection of rat against B. pertussis infection induced with the 1/5 or 1/10 human dose ID immunization compared with 1HD dose IM immunization or PBS. Rats were challenged 4 weeks after the third immunization by aerosol with B. pertussis (1 × 10^11^CFU/ml) over 30 min. Results were shown as the number of bacteria recovered from the rat lungs at different time points, expressed as the log10 of the means ± SEM (*n* = 4 rats per group per time-point) of the CFUs in **(A)** lung, data was analyzed using a two-way ANOVA model with Tukey’s multiple comparisons test. *P < 0.05, **P < 0.01, ****P < 0.0001 vs 1/5D or 1/10D ID and ^#^*P* < 0.05, ^####^*P* < 0.0001 vs 1D IM. The CFUs in **(B)** trachea and **(C)** nasal wash was shown in figure, expressed as the log10 of the means ± SEM (*n* = 4 rats per group per time-point). *P < 0.05, **P < 0.01, ***P < 0.001 vs PBS and ^#^*P* < 0.05, ^##^*P* < 0.01 vs 1D IM group. **(D–F)** show the areas under the bacterial clearance curves corresponding to the curves in panels **(A–C)**.

Compared with that of the control group, the bacterial colonization of all immunization routes was significantly lower, while the best protection was observed in ID immunized rats (1/5 dose or 1/10 dose) (Figures 5A,D). The areas under the bacterial clearance curve in the lung showed that the bacterial load was lower in rats immunized intradermally with a 1/5 dose (5.345) or 1/10 dose (4.241) than in the IM group administered the full-dose vaccine (14.852) or PBS (59.214). In addition, ID immunization also induced faster lung bacterial clearance (Figure 5A); bacteria in the lungs of the 1/5D ID group were completely cleared on day 10, no rebound was seen by day 21, and the 1/10D ID group also showed complete clearance on the 14th day after *B. pertussis* challenge, but the 1D IM group did not show complete clearance until the 21st day. In trachea and nasal wash fluids (Figures 5B,C,E,F), the CFUs of rats in the 1/5D ID and 1/10D ID groups were significantly lower than those of rats in the PBS and 1D IM groups in the early stage of infection (2 days after challenge), indicating that ID immunization prevented bacterial colonization effectively. In summary, compared to IM immunization, low-dose ID immunization induced more effective lung and upper respiratory tract protection against *B. pertussis* infection and faster clearance of bacteria in the lungs.

### Cytokine Signatures Suggest Increased Th1, Th2, and Th17 Responses After Intradermal Immunization

**FIGURE 6:**
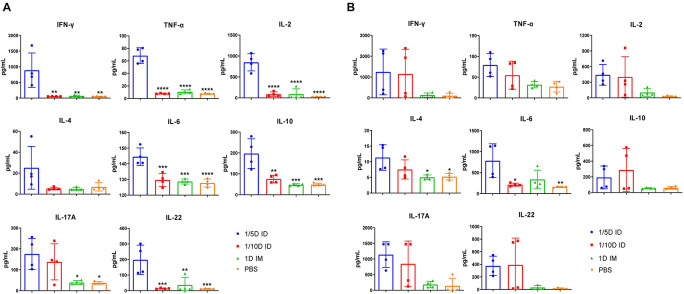
Th1/Th17-related cytokines have increased significantly after intradermal immunization. Four weeks after the last immunization, **(A)** spleen and **(B)** lung lymphocytes were isolated. Cells were cultured at a concentration of 2 × 10^6^/ml at 37°C with 5% CO2 and stimulated with the antigens PT (1 μg/ml), FHA (1 μg/ml), and PRN (1 μg/ml). Later, the supernatant was collected and detected the content of multiple cytokines in the supernatant by ELISA method, and reflects the type of immune response indirectly, where IL-2, IFN-γ, TNF-α corresponds to Th1 cell response; IL-17A, IL-22, IL-6 corresponds to Th17 cell response; IL-4, IL-10 represents Th2 cell response and inflammatory inhibition response, respectively. Results shown as pg/mL of the mean ± SEM. Data were analyzed using an one-way ANOVA model with Tukey’s multiple comparisons test. *P < 0.05, **P < 0.01, ***P < 0.001, and ****P < 0.0001 vs 1/5D ID group (*n* = 4).

After incubation with PT, FHA, PRN for 72 h, cytokines in the supernatant of lung and spleen lymphocytes cultures were tested 4 weeks after the third dose of the vaccine. The differences in cytokine levels in the lungs and spleen were observed among the ID and IM groups. In spleen lysates, the levels of cytokines associated with the Th1 response (IFN-γ, TNF-α, and IL-2) and Th17 response (IL-17A, IL-6, and IL-22) were significantly increased in the 1/5D ID group compared with the 1D IM or PBS group. In addition, the production of the Th2-related cytokines (Raeven et al., 2020) IL-10 and IL-4 were increased in spleen lymphocytes of 1/5D ID compared to 1D IM or PBS-rat (Figure 6A). In lung lysates, intradermal immunization caused a slight increase in Th1/2/17-related cytokine levels, but there was no significant difference; IL-4 levels were significantly higher than that in the 1D IM group (Figure 6B). Overall, these results demonstrate that intradermal immunization with 1/5 dose of DTaP-sIPV may cause Th1/Th2/Th17-related cytokine responses.

### Pulmonary Transcriptomic Signatures

**FIGURE 7:**
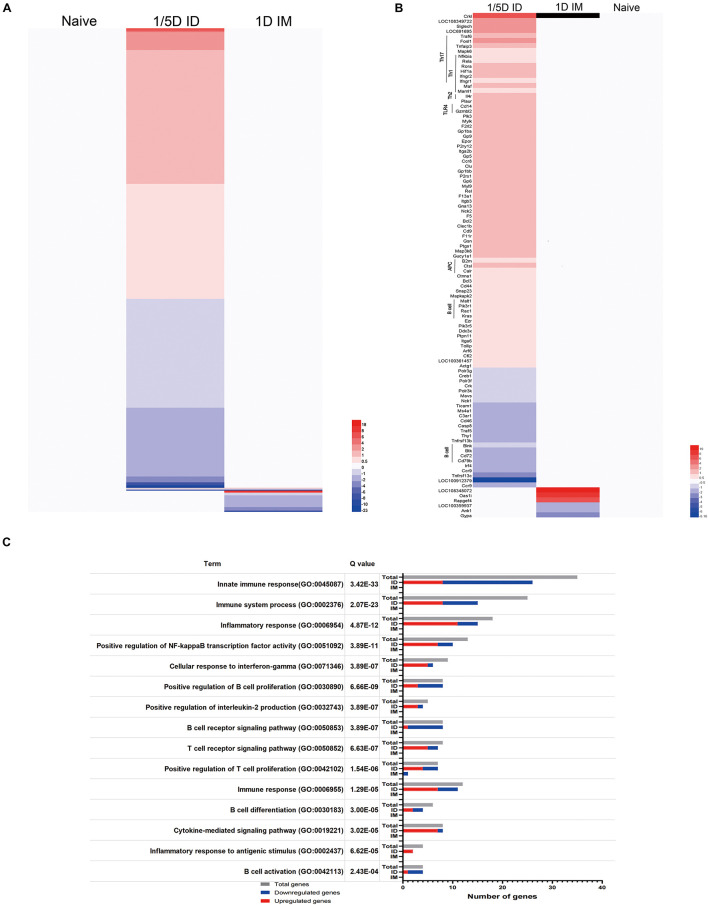
Pulmonary transcriptomic signatures of ID or IM immunization compared with naïve rat. Gene expression in 1/5D ID and 1D IM rats was compared with naive rats [fold ratio (FR) > 1.5, *Q* value < 0.05, corrected by the Bonferroni test]. **(A)** A total of 1,026 genes upregulated (red) or downregulated (blue) are visualized in heatmaps (mean of *n* = 3). Genes not surpassing a fold change of 1.5 are shown as basal level (white). **(B)** Immune related genes, including association with distinct T helper (Th) subsets, Toll like receptor (TLR), antigen presenting cells (APC) and B cell response. **(C)** Over-representation analysis on all 1,026 genes revealed the involvement of specific Gene Ontology Biological Process (GO-BP) terms with corresponding total amount of genes (gray) and upregulated (red) and downregulated (blue) genes.

Transcriptome sequencing analysis of lungs from rats in the 1/5D ID, 1/10D ID, and 1D IM groups revealed 1,026 differentially expressed genes (DEGs; *Q* value < 0.05, FR > 1.5) when compared with naïve rat (Figure 7A and Supplementary Figure 4). Of these DEGs, 576 genes were upregulated and 405 were downregulated exclusively after 1/5D ID immunization, while only seven genes were upregulated and 44 were downregulated exclusively after 1D IM immunization. There were only two upregulated genes and four downregulated genes in both 1/5D ID and 1D IM rats. Then, we performed OR analysis of all DEGs, and the results showed that they were enriched in 454 Gene Ontology Biological Process (GO-BP) terms and 52 Kyoto Encyclopedia of Genes and Genomes (KEGG) pathways. A selection of immunologically relevant terms is shown in Figures 7B,C.

Most of the genes upregulated in 1/5D ID rats were enriched in T cell-related terms rather than B cell-related terms in GO-BP (Figure 7C). The significant upregulation of Rela, Rora, Hif1a, Fosl1, Ptpn11, Ifngr1, and Ifngr2 expression in ID rats indicates a shift to a Th1/Th17 response. The antigen presentation and processing process were also shown to be activated, with upregulation of B2m, Ctsl, Calr, and Pdia3. In addition, the expression of genes related to the TLR4 (Tollip, Cd14), PI3K (Pik3r1, Pik3r5) and NF-κB (Nfkbia) pathways were upregulated (Figure 7B). In general, compared with IM immunization and naive rats, ID immunization caused a significant upregulation of immune genes related to the Th1/Th17 response and antigen processing and presentation.

## Discussion

Due to the presence of antigen-presenting cells in the epidermis and dermis, ID immunization is an ideal method of immunization. A new type of hollow microneedle named the MicronJet600 has a special structure and can achieve more accurate and efficient ID immunization (Levin et al., 2015). In this study, the effect of ID immunization with a low dose of DTaP-sIPV with the MicronJet600 was evaluated in rats and compared with that of full-dose IM immunization. To our knowledge, this study is the first to assess the immunogenicity and dose-sparing effect of DTaP-sIPV after ID immunization.

First, we investigated the dose-sparing effect on both sIPV and DTP antibody responses. With 10-100 times of the antigen formulation of polio type 1-3, IPV is approximately 20 times more expensive than OPV, which limits the supply of IPV available to satisfy the requirements of the polio endgame strategy worldwide (Okayasu et al., 2017). Fractional IPV as an antigen-sparing technique could be a key strategy to reduce the cost of IPV and stretch its limited supply. In fact, several studies (Schipper et al., 2016; Snider et al., 2019) have evaluated the dose-sparing effect of cIPV and indicated that fIPV (a 1/5 human dose of IPV) can cause the same immune effect as full-dose cIPV IM immunization. In our study, which is the first investigation of fractional dose administration of sIPV in rats, we observed no difference in vaccine responses for all serotypes after ID immunization with a 1/5 dose of DTaP-sIPV compared with full-dose IM immunization; in other words, the dose-sparing effect increased by fivefold. We also examined antibody levels at 6 months after immunization was completed and showed similar antibody levels in the 1/5D ID and the 1D IM groups. This may imply that intradermal immunization also induced a long-lasting humoral immune response, which induced comparable long-term protection compared to full-dose muscle immunity. However, longer observations and more tests for immune memory indicators are still needed to support these assumptions. In addition to the 1/5 dose, the 1/10 dose did not show lower titers of the IPV NAbs for serotypes 1 and 2 of poliovirus than those in the 1D IM group. The NAb titer for serotype 3 was lower in the 1/10D ID group than in the 1D IM group. A possible explanation is that when the corresponding antigen dose is reduced, the NAb level for serotype 3 decreased more significantly compared to the other two serotypes (Kraan et al., 2014; Wan et al., 2018), and the decreased Nab level caused by dose reduction could not be offset by improved immunogenicity induced by ID immunization in 1/10D ID group.

In addition, because of the high immunogenicity of DTaP vaccine, only a 1/20 human dose can induce effective protection against *B. pertussis* infection in mice (Boehm et al., 2018). Thus, to evaluate the differences between ID and IM immunization, we reduced the dose of DTaP vaccines in all groups by an equal proportion. The 1D IM group used a 1/4 human dose of the DTaP vaccine, so the corresponding 1/5D ID group used a 1/20 human dose, and the 1/10D ID group used a 1/40 human dose. According to a previous study (Boehm et al., 2018), immunization with a 1/40 human dose failed to provide effective protection against *B. pertussis* infection in a mouse model. However, in this study, ID immunization with 1/40 human dose of DTaP induced effective *B. pertussis* infection protective response, including nasal, tracheal and lung colonization, but this response was weaker than that observed in the 1D IM group. In view of the protective effect of the 1/5 human dose in mice, the dose-sparing effect caused by ID administration could be fivefold higher, and for subsequent clinical studies, the DTaP dose should start with 1/5 dose.

According to multiple clinical studies on ID immunity of fractional doses of cIPV (Snider et al., 2019; Bandyopadhyay et al., 2021), ID immunization with two doses of 1/5 dose of cIPV can induce the same levels of NAbs as full-dose IM immunization. In the present study, immunization with the 1/5 dose of DTaP-sIPV ID induced the same levels of DTaP-related antibodies and poliovirus NAbs as IM immunization. However, comparing to the first two doses, the third dose did not cause a significant increase in the levels of any antibody except for Nabs for type 2 poliovirus antibodies in the 1/5D ID group. Furthermore, the three-dose immunization program even caused a significant decreasing in antibodies for FHA and PRN antibodies in the 1/10D ID and 1D IM groups. A possible explanation for this phenomenon was that the interval between the two vaccinations was only 4 weeks, which may be too short to fully stimulating the immune memory response. Studies (Anand et al., 2017; Bandyopadhyay et al., 2021) have shown that longer intervals between doses are directly related to stronger immunogenicity, which may also apply to DTaP-sIPV vaccines. Snider et al. (2019) compare fractional dose cIPV with full doase cIPV and cIPV + fIPV vaccination and indicated that two fIPV does delivered at 6 and 14 weeks of age induced higher type 1 and type 3 poliovirus NAbs than three fIPV doses. Bandyopadhyay et al. (2021) further demonstrate that two doses of f-cIPV administered at 14 and 36 weeks could provide adequate immunity against poliovirus type 1 and type 2. Thus, we speculate that two doses administered at a longer interval than used in this study may induce a better immune response in future experiments. The current observations will help to develop optimal immune procedures for clinical trials of DTaP-sIPV intradermal immunization. This will help to save antigen and improve immunogenicity.

In addition to the humoral response, we observed a distinct cellular immune response to aPV vaccine components in rats immunized intradermally with the MicronJet600. Injection with the MicronJet600 is characterized by an intradermal bleb or wheal, which has been considered acceptable during intradermal injection (Maluf, 2002) and prolongs the contact time between antigen and DC or T cells. Some studies (Kundig et al., 1996; Bachmann et al., 2006) showed that this prolonged antigen exposure changed the immune profile and induces a superior immune response.

Th1 and Th17 cell responses play a key role in immune protection against *B. pertussis*, killing intracellular bacteria by recruiting macrophages and neutrophils to the lungs (Ross et al., 2013), which is related to the efficiency of *B. pertussis* clearance in the lungs of immunized mice or human being (Mascart et al., 2007; Klein et al., 2012; Ross et al., 2013; Warfel et al., 2014). In this study, faster lung clearance was observed in ID-immunized group after *B. pertussis* aerosol challenge and detection of cytokines levels of the 1/5D ID group showed a significant increase in IL-2, IFN-γ, TNF-α, IL-17, and IL-22 in spleen lymphocytes compared to other groups before aerosol challenge, suggesting that intradermal immunization may cause an effective Th1/17 cell immune responses, which play a key role in the clearance of *B. pertussis*. In addition, 1/5 dose of ID immunization also caused an increase in Th2-related cytokines, which may be associated with the ID immunization dose-sparing effects for antibody response. Thus, we deduced that 1/5 dose of ID immunization may cause Th1/Th2/Th17 immune responses, similar to the results of other *B. pertussis* candidate vaccine studies (Bottero et al., 2016; Raeven et al., 2020), which are associated with better prevention against *B. pertussis* and faster clearance. However, due to the limitations of the number of experimental animals and the effects of individual differences, present study only provides a clue for the potential advantage of the ID immunization. Further ID immunological studies in other experimental animal models, such as non-human primate model or others, should be performed.

Studies in IFN-γR-/- mice (Ross et al., 2013) have demonstrated that IFN-γ can activate macrophages to eliminate intracellular bacteria and play an important role in immune protection against *B. pertussis* (Higgins et al., 2006) and that IL-17 exerts antibacterial effects by recruiting neutrophils to the site of infection and promoting the synthesis of antibacterial peptides (Ye et al., 2001; Khader et al., 2007). In the present study, the expression of the IFN-γ receptor genes Ifngr1 and Ifngr2 was significantly increased in the lungs after immunization. It has been reported that rapid co-polarization of IFNGR with the TCR occurs within the developing immunological synapse, suggesting an important impact of the physical co-polarization of critical receptors on naive T-helper lymphocyte (Maldonado et al., 2004). Ptpn11 is the gene that encodes for SHP-2 and it was crucial for the induction of interleukin 1b (IL-1b), IL-6 and IL-23 and responses of the Th17 subset of helper T cells in controlling infection (Deng et al., 2015). The upregulation of Rela, Rora, Hif1a, Nfkbia, Pik3r1, and Pik3r5 indicates the involvement of PI3K and the NF-κB pathway in intradermally immunized rats (Jetten, 2004; Ivanov et al., 2006; Raeven et al., 2018), which also play a critical role in Th17 differentiation. The expression of B2m, Ctsl, Calr, and Pdia3 suggests efficient antigen processing and presentation process, and the upregulation of Rac1, and Cd14 indicats the involvement of TLR2 and TLR4, which are essential for Th1/Th17 responses elicited by wPVs or bacterial infection (Higgins et al., 2006; Reynolds et al., 2010; Brummelman et al., 2015). Moreover, low-dose ID immunization can induce more effective lung and upper respiratory tract protection against *B. pertussis* infection and faster clearance of bacteria in the lungs. Thus, we deduced that ID immunization can induce efficient Th1 and Th17 cell responses to clear *B. pertussis* infection.

However, there are still some limitations in this study. Firstly, the time point for lung cytokine detection was set 4 weeks after the third vaccination, which was later than the time of peak immune response in the lungs (Raeven et al., 2018). Therefore, setting multiple detection time points within the weeks after each immunization may result in more significant differences in the lung. Second, based on these results and existing studies (Anand et al., 2017; Bandyopadhyay et al., 2021), it can be inferred that the interval between the two vaccinations in this study can also be appropriately extended to 6–8 weeks to achieve better immunity after the boosters. In general, we conducted a systematic study of the immune status of rats immunized with intradermally low-dose DTaP-sIPV with the MicronJet600, which confirmed that hollow microneedles are an excellent tool for intradermal vaccines. The same poliovirus- and pertussis-related antigen antibody levels were observed after intradermal immunization with a 1/5 dose and after full-dose intramuscular immunization. In addition, effective DTaP-sIPV ID immunization may induce mixed Th1, Th2, and Th17 responses in rats, thereby providing effective protection and rapid elimination of *B. pertussis* bacterial infection. This obvious dose-sparing effect may be used to increase DTaP immunogenicity and expand the current IPV supply. This study shows that intradermal immunization of DTaP-sIPV with MicronJet600 elicit superior immunity and significant dose-sparing effect, which highlight its potential as a new DTaP-sIPV vaccine strategy. This obvious dose-sparing effect may be used to increase DTaP immunogenicity and expand the current IPV supply. Further studies on the 2-dose 1/5D ID schedule with longer intervals could be performed in rats and non-human primates.

## Data Availability Statement

The sequences data reported in this study was archived in the Sequence Read Archive (SRA) with the accession numbers: SRR15347873, SRR15347872, SRR15347871, SRR15347870, SRR15347869, SRR15347868, SRR15347867, SRR15347866, SRR15347865, SRR15347864, SRR15347863, and SRR15347862.

## Ethics Statement

The animal study was reviewed and approved by The Experimental Animal Ethics Committee of Institute of Medical Biology, Chinese Academy of Medical Sciences.

## Author Contributions

LS and MS conceived and designed the study. WZ, WJ, and MZ performed the study. YM, QG, XW, and LC provided the necessary research reagents, animals, and technical expertise. WZ and JL analyzed the data. LS, MS, and WZ wrote the manuscript. All authors have read and approved the final manuscript.

## Conflict of Interest

The authors declare that the research was conducted in the absence of any commercial or financial relationships that could be construed as a potential conflict of interest.

## Publisher’s Note

All claims expressed in this article are solely those of the authors and do not necessarily represent those of their affiliated organizations, or those of the publisher, the editors and the reviewers. Any product that may be evaluated in this article, or claim that may be made by its manufacturer, is not guaranteed or endorsed by the publisher.

## Footnotes

1
http://www.geneontology.org/

2
https://www.kegg.jp/

3
https://en.wikipedia.org/wiki/Hypergeometric_distribution
