# Supplementary figures and images for: Dose-Sparing Intradermal DTaP-sIPV Immunization With a Hollow Microneedle Leads to Superior Immune Responses
Source: Front Microbiol. 2021 Oct 25;12:757375. doi: 10.3389/fmicb.2021.757375 (PMC8573275; doi:10.3389/fmicb.2021.757375)

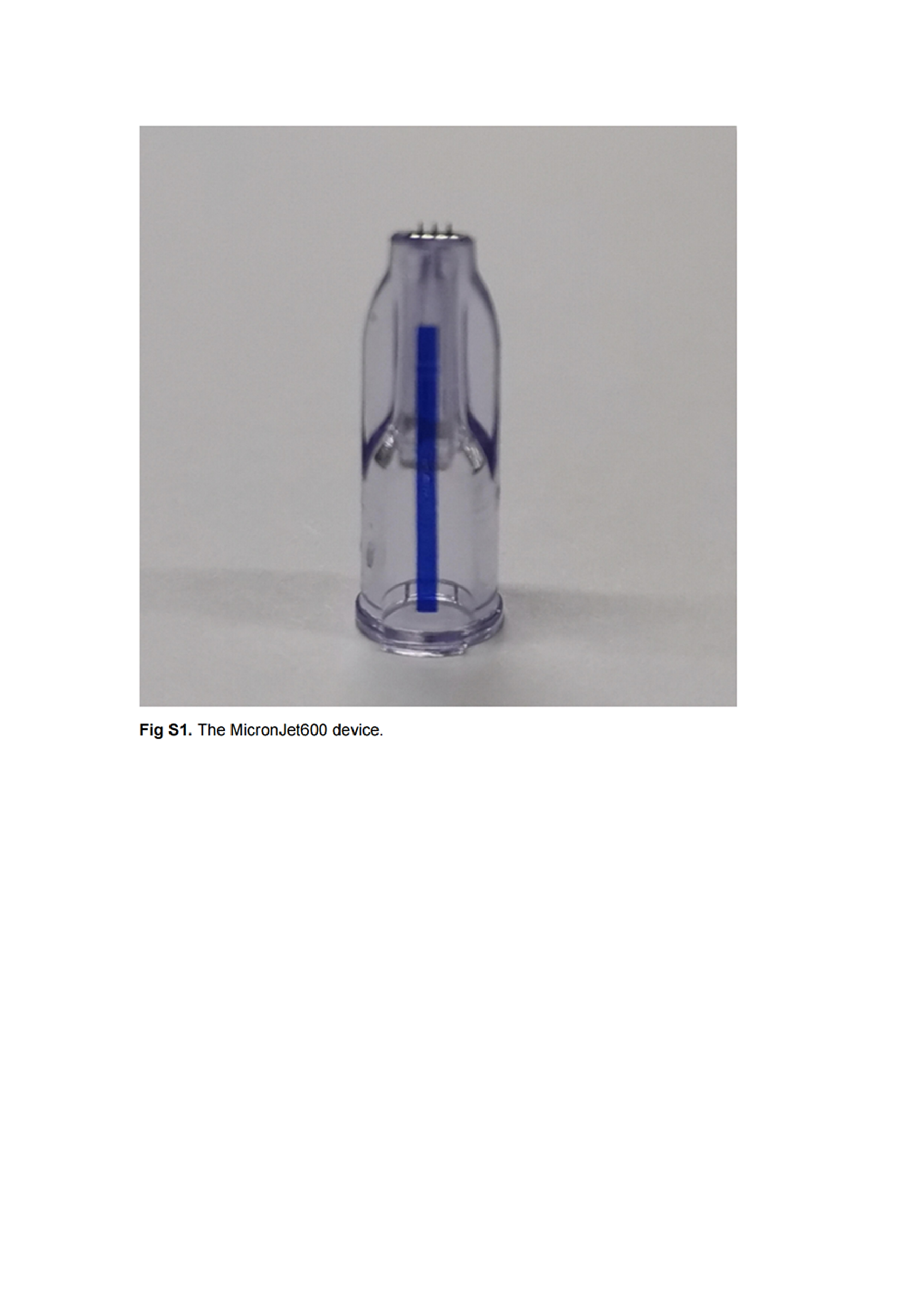

Supplement: Supplementary file 1 [file Image_1.TIF]

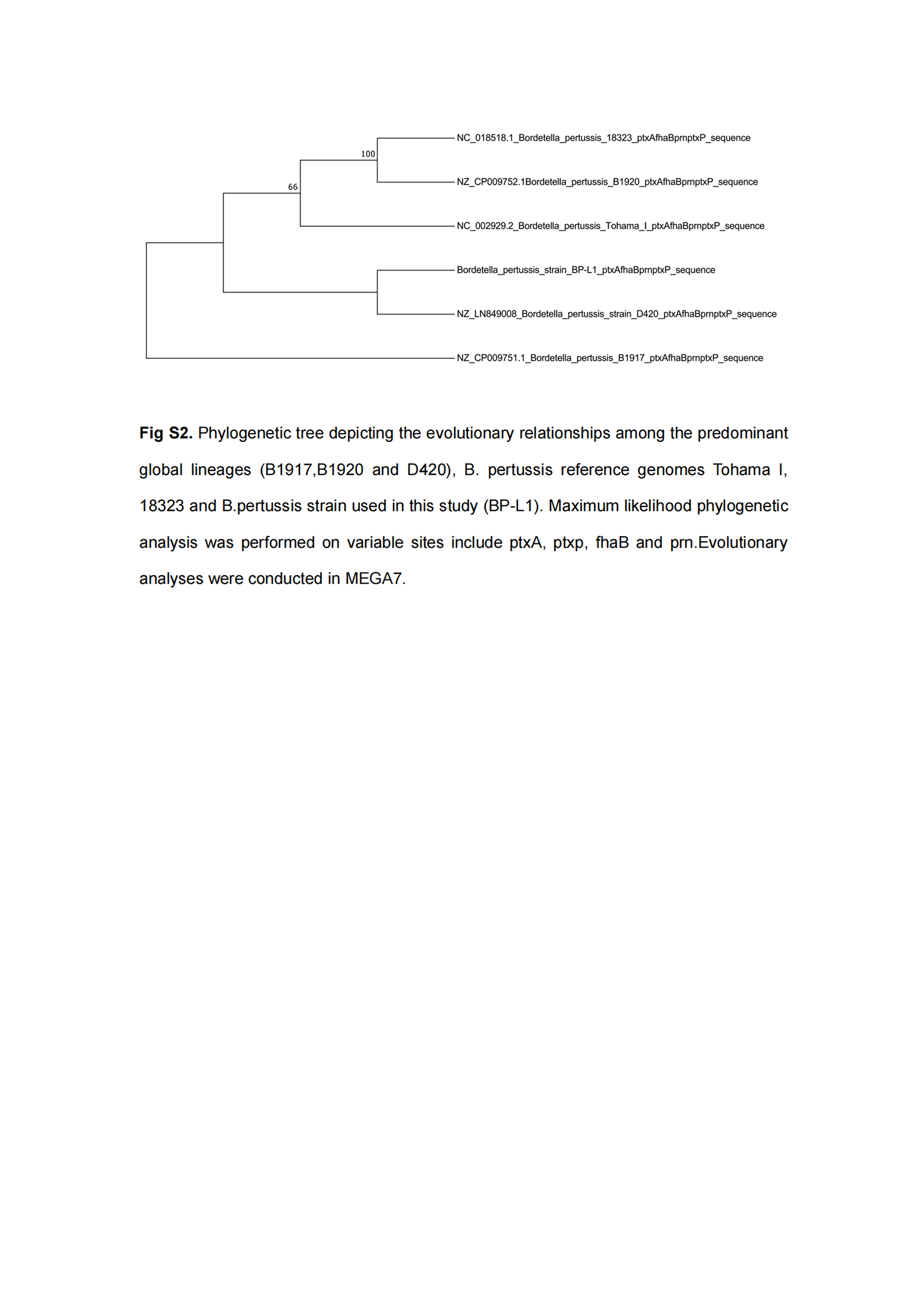

Supplement: Supplementary file 2 [file Image_2.TIF]

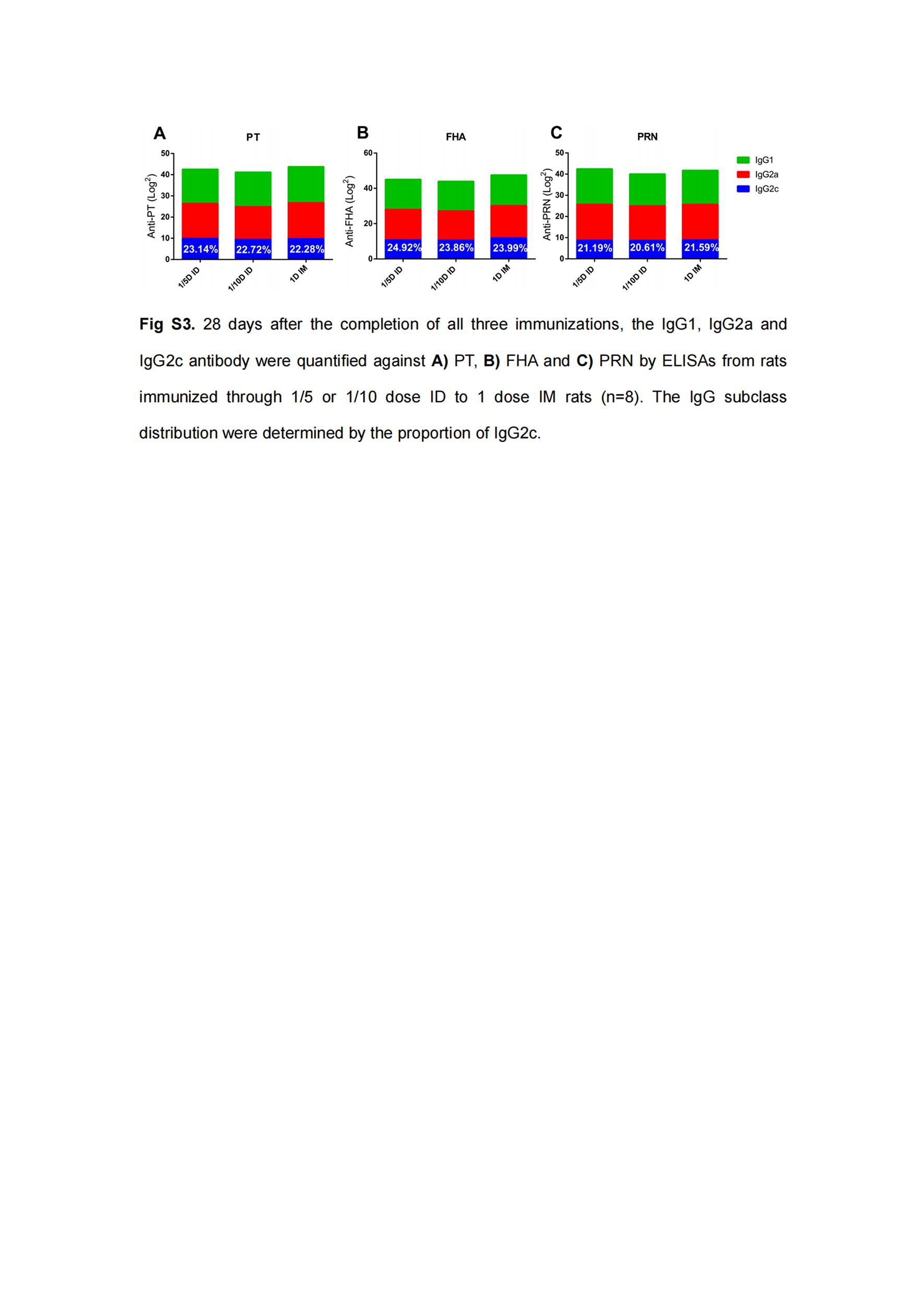

Supplement: Supplementary file 3 [file Image_3.TIF]

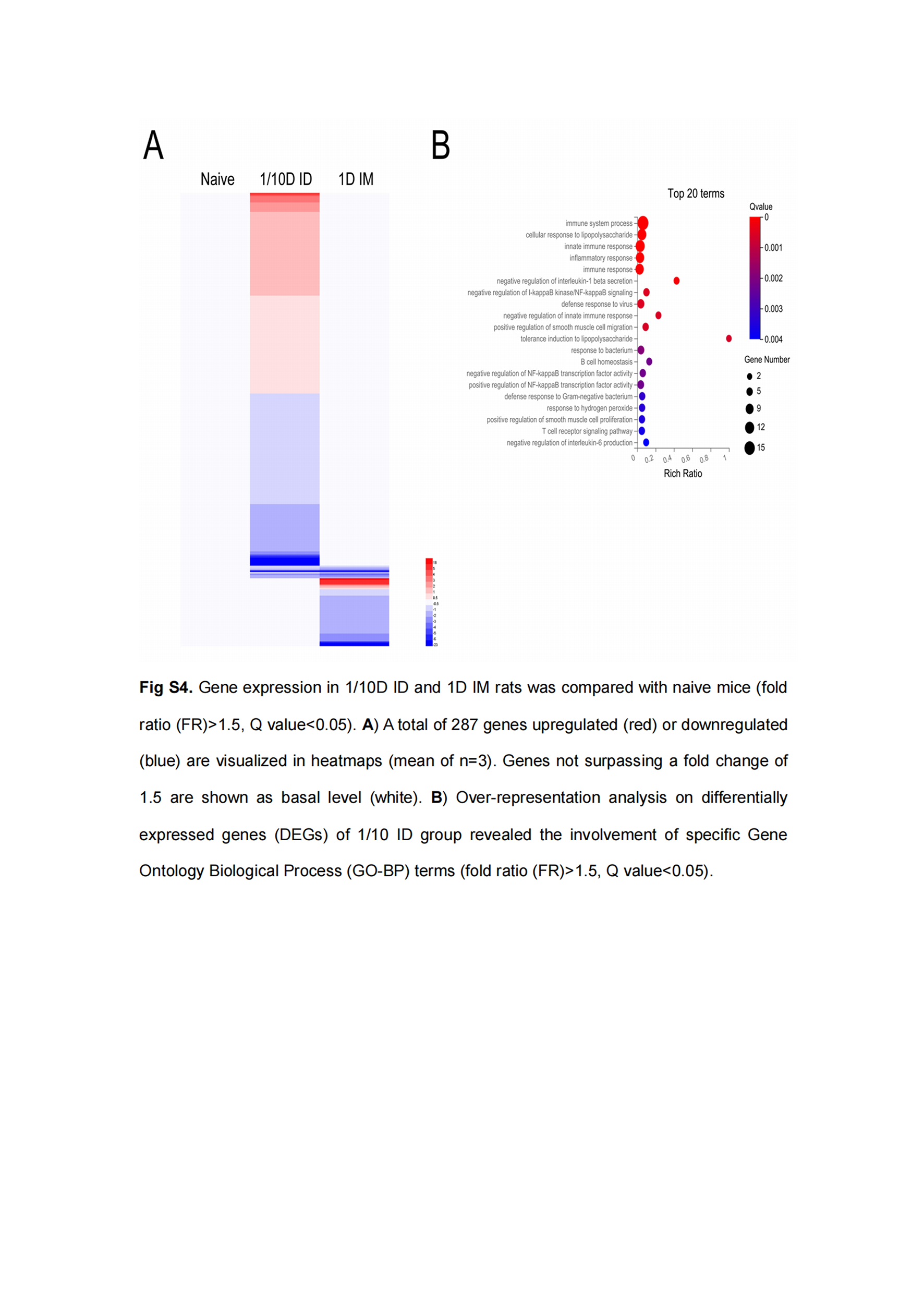

Supplement: Supplementary file 4 [file Image_4.TIF]
